# Sex differences in grey matter networks in dementia with Lewy bodies

**DOI:** 10.21203/rs.3.rs-2519935/v1

**Published:** 2023-01-30

**Authors:** Annegret Habich, Javier Oltra, Christopher G Schwarz, Scott A Przybelski, Ketil Oppedal, Anna Inguanzo, Frédéric Blanc, Afina W Lemstra, Jakub Hort, Eric Westman, Val J Lowe, Bradley F Boeve, Thomas Dierks, Dag Aarsland, Kejal Kantarci, Daniel Ferreira

**Affiliations:** Division of Clinical Geriatrics, Center for Alzheimer Research, Department of Neurobiology, Care Sciences and Society, Karolinska Institutet, Stockholm, Sweden; Medical Psychology Unit, Department of Medicine, Institute of Neurosciences, University of Barcelona, Barcelona, Spain; Department of Radiology, Mayo Clinic, Rochester, USA; Quantitative Health Sciences, Mayo Clinic, Rochester, MN, US; Center for Age-Related Medicine, Stavanger University Hospital, Stavanger, Norway; Division of Clinical Geriatrics, Center for Alzheimer Research, Department of Neurobiology, Care Sciences and Society, Karolinska Institutet, Stockholm, Sweden; Day Hospital of Geriatrics, Memory Resource and Research Centre (CM2R) of Strasbourg, Department of Geriatrics, Hopitaux Universitaires de Strasbourg, Strasbourg, France; Department of Neurology and Alzheimer Center, VU University Medical Center, Amsterdam, Netherlands; Motol University Hospital, Prague, Czech Republic; Division of Clinical Geriatrics, Center for Alzheimer Research, Department of Neurobiology, Care Sciences and Society, Karolinska Institutet, Stockholm, Sweden; Department of Radiology, Mayo Clinic, Rochester, USA; Department of Neurology, Mayo Clinic, Rochester, USA; University Hospital of Psychiatry and Psychotherapy Bern, University of Bern, Bern, Switzerland; Department of Old Age Psychiatry, Institute of Psychiatry, Psychology & Neuroscience, King’s College London, London, UK; Department of Radiology, Mayo Clinic, Rochester, USA; Division of Clinical Geriatrics, Center for Alzheimer Research, Department of Neurobiology, Care Sciences and Society, Karolinska Institutet, Stockholm, Sweden

**Keywords:** dementia with Lewy bodies, sex differences, grey matter networks

## Abstract

**Objectives:**

Sex differences permeate many aspects of dementia with Lewy bodies (DLB), including epidemiology, pathogenesis, disease progression, and symptom manifestation. However, less is known about potential sex differences in patterns of neurodegeneration in DLB. Here, we test whether grey matter networks also differ between female and male DLB patients. To assess the specificity of these sex differences to DLB, we additionally investigate sex differences in healthy controls (HCs).

**Methods:**

A total of 119 (68.7 ± 8.4 years) male and 45 female (69.9 ± 9.1 years) DLB patients from three European centres and the Mayo Clinic were included in this study. Additionally, we included 119 male and 45 female age-matched HCs from the Mayo Clinic. Grey matter volumes of 58 cortical, subcortical, cerebellar, and pontine brain regions derived from structural magnetic resonance images were corrected for age, intracranial volume, and centre. Sex-specific grey matter networks for DLB patients and HCs were constructed by correlating each pair of brain regions. Network properties of the correlation matrices were compared between sexes and groups. Additional analyses were conducted on W-scored data to identify DLB-specific findings.

**Results:**

Networks of male HCs and male DLB patients were characterised by a lower nodal strength compared to their respective female counterparts. In comparison to female HCs, the grey matter networks of male HCs showed a higher global efficiency, modularity, and a lower number of modules. None of the global and nodal network measures showed significant sex differences in DLB.

**Conclusions:**

The disappearance of sex differences in the structural grey matter networks of DLB patients compared to HCs may indicate a sex-dependent network vulnerability to the alpha-synuclein pathology in DLB. Future studies might investigate whether the differences in structural network measures are associated with differences in cognitive scores and clinical symptoms between the sexes.

## Introduction

Traditionally, dementia with Lewy bodies (DLB) has been considered a male-predominant disease. Autopsy results almost unequivocally support this notion (Nelson et al. 2010). However, the sex distribution of DLB patients diagnosed clinically is more ambiguous. While most European and North American cohorts are dominated by male DLB patients (Perez et al. 2010; Fereshtehnejad et al. 2013; Savica et al. 2013; Kane et al. 2018), a systematic review on clinical studies as well as French and Chinese cross-sectional studies pointed at a less consistent sex distribution in DLB (Jones and O’Brien 2014; Mouton et al. 2018; Gan et al. 2022).

Other studies demonstrated that differences in female and male DLB patients permeate numerous aspects of the disease presentation. In that regard, a pathology study showed that the Lewy body pathology in male DLB patients tended to occur at an earlier age and was confined to brain stem and limbic system (Fujimi et al. 2008). In contrast, female DLB patients tended to manifest Lewy body pathology later in life combined with a more pronounced neocortical spread, suggesting a more aggressive disease course. The occurrence of an accelerated disease course in female DLB patients was corroborated by a recent in-vivo study (van de Beek et al. 2020). Therein, female DLB patients exhibited more abnormal concentrations of α-synuclein in cerebrospinal fluid, were older, had a shorter duration of cognitive complaints, and displayed more psychiatric and cognitive symptoms than male DLB patients. Additionally, sex differences have been found regarding core clinical features of DLB. Whereas male DLB patients more often present with REM-sleep behavioural disorder (RBD) and parkinsonism (Bayram et al. 2021; Utsumi, Fukatsu, and Hara 2021), psychiatric symptoms are more frequent in female DLB patients (Chiu et al. 2018; Tsunoda et al. 2018; Utsumi, Fukatsu, and Hara 2021) but compare (Bayram et al. 2021). Especially hallucinations, both visual and auditory, are more frequent in female DLB patients and the content of the hallucinations also differs between the sexes (Chiu et al. 2018; Tsunoda et al. 2018).

Since there is currently no available topographical biomarker for the spread of α-synuclein pathology in-vivo, its association with sex differences in incidence rates or clinical features cannot be assessed directly. Yet, the transneuronal spread of the pathology (Masuda-Suzukake et al. 2013; Warren et al. 2013; Wakabayashi 2020) can potentially be tracked through the network by its impact on distinct brain regions using magnetic resonance imaging (MRI). In this regard, intrinsic structural networks have been shown to shape the pattern of atrophy in patients with Parkinson’s disease (PD), another α-synucleinopathy (Zeighami et al. 2015; Tremblay et al. 2021). The observed higher level of atrophy in regions surrounding the substantia nigra (Yau et al. 2018), which is hypothesized to be affected early during the α-synuclein spread, provides a possible link to the α-synuclein pathology. Hence, investigating the global pattern of atrophy via grey matter networks on MRI data may not only indicate the spread of Lewy body pathology but also which affected brain regions may underlie the symptoms. Using a graph theoretical approach, a previous study found differences in large-scale structural grey matter networks between DLB patients and healthy controls, yet it did not investigate sex differences (Nicastro et al. 2021). In contrast, two MRI studies investigated sex differences in regional and lobar atrophy but did not inspect grey matter networks (Abdelnour et al. 2020; Ballmaier et al. 2004). In these studies, males had a greater grey matter loss compared to female DLB patients, especially in frontal regions. Hence, sex differences in grey matter networks of DLB patients remain to be investigated.

The present study aimed to assess the differences in grey matter network topologies between female and male DLB patients using graph theoretical analyses on volumetric measures from structural MRI. We also aimed to compare the findings to network differences in healthy elderly controls. We hypothesised that sex differences in network topologies differ between DLB patients and HCs, thus pointing towards DLB-specific sex differences. Structural deterioration connected to DLB may redirect volume reductions experienced during normal ageing. Hence, we further aimed to disentangle the two processes and extract DLB-specific sex differences in grey matter networks by constructing grey matter networks on W-scored DLB data, thus removing the effects of normal ageing.

## Methods

### Participants

In this multicentre study, we used DLB patient data (n = 164, 45 female) from three centres of the E-DLB consortium (Prague, Strasbourg, Amsterdam) (Oppedal et al. 2019) as well as the Mayo Clinic DLB cohort (Kantarci et al. 2017). Probable DLB patients were diagnosed according to the 2005 International Consensus Criteria (McKeith et al. 2005). Patients were further characterized by the presence or absence of the core clinical features of DLB (parkinsonism, visual hallucinations, cognitive fluctuations, and REM sleep behaviour disorder), which were recorded at baseline. Additionally, performance in the Mini-Mental State Examination (MMSE) was assessed as a measure of global cognition. To assess the presence of Alzheimer’s disease (AD) co-pathology, AD biomarkers were measured in cerebrospinal fluid in E-DLB centres and with positron emission tomography at the Mayo Clinic as described elsewhere (Ferreira et al. 2020). Using centre-specific cut-points, positivity in both β-amyloid and tau biomarkers was interpreted as the presence of an AD co-pathology. Participants were excluded when they presented with any of the following: presence of acute delirium, terminal illness, previous stroke, psychotic or bipolar disorder, craniocerebral trauma, and recent diagnosis of a major somatic illness. For comparison, we included 164 sex- and age-matched, cognitively unimpaired participants from the Mayo Clinic Study of Aging (MCSA, (Roberts et al. 2008)).

The study was approved by the local ethics committee at each E-DLB centre and the Mayo Clinic Institutional Review Board. In compliance with the Declaration of Helsinki, all participants or appropriate surrogates provided written informed consent prior to their participation in the study.

### MRI Data Acquisition

A high-resolution 3D T1-weigthed magnetization-prepared rapid gradient-echo (MPRAGE) sequence was acquired in all four centres included in this study. At the Day Hospital of Geriatrics, Memory Resource and Research Centre (CMRR, Strasbourg, France), the Mayo Clinic (Rochester, US), and the VU University Medical Center (VUmc, Amsterdam, the Netherlands) images were acquired at a magnetic field strength of 3 T whereas the Motol University Hospital (Prague, Czech Republic) used a 1.5 T scanner.

### MRI Preprocessing

All MRI data were processed at the Mayo Clinic. Using Advanced Normalization Tools (ANTs; (Avants et al. 2008)), the Mayo Clinic Adult Lifespan Template (MCALT; https://www.nitrc.org/projects/mcalt/) atlas was registered to individuals’ native MPRAGE space. T1-MPRAGE images were then tissue-class segmented, using the unified segmentation algorithm in SPM12 (Wellcome Trust Center for Neuroimaging, London, UK) run in Matlab (Mathworks, Natick, MA) with priors and settings from the MCALT. Following MCALT parcellation, we obtained the volumes of 58 grey matter regions-of-interest (ROIs), consisting of 41 cortical (sum of both hemispheres), 6 subcortical, 9 cerebellar, and 2 brainstem ROIs, for each participant.

Additionally, the total intracranial volume (TIV) was calculated as the sum of tissue probabilities of grey matter, white matter, and cerebrospinal fluid segmentations.

### Network Construction and Analysis

Grey matter networks were constructed from the volumetric data of 58 ROIs. For aim 1, ROI data of the DLB patients was adjusted for TIV and centre using multiple linear regression prior to network construction. As data for the healthy controls were collected at a single centre, ROIs of healthy controls were only corrected for TIV.

To identify DLB-specific sex differences in the grey matter network structure, we regressed out the expected effect of TIV, age, and sex by calculating W-scores. For each ROI, the following formula was applied:

$$W-{\text{score}}_{\text{DLBpatient}}=\frac{{\text{rawvalue}}_{\text{DLBpatient}}-{\text{expectedvalue}}_{\text{control}}\left(\text{forpatient}\prime \text{sTIV},age,sex\right)}{{\text{SDofresiduals}}_{\text{controls}}}$$

W-scores were additionally corrected for centre, using linear regression. The resulting residuals were used for the construction of sex dependent grey matter networks in the DLB patients.

As a result of these three procedures, separate grey matter networks were constructed for each sex for DLB patients, healthy controls, and W-scored data. Therein, nodes correspond to the residual volumes of the 58 ROIs and edges represent the Pearson correlation coefficients between each node pair. Negative correlations and self-connections along the diagonal were removed in the weighted correlation matrices.

We calculated several measures to define the centrality, integration, and segregation of the grey matter networks, both on the global (averaging across all nodes) and nodal (concerning single nodes) level (Rubinov and Sporns 2010). Nodal strength, (the sum of all connections of a node), a measure of centrality, was calculated on the weighted correlation matrices. All other network measures were calculated on unweighted binarized networks, which were constructed by thresholding networks at densities between 20–50% in steps of 1%. This procedure ensured that the network was neither disconnected (network density < 20%) nor exhibited a random topology (network density > 50% with small-world index ~ 1). Sex differences in grey matter network parameters, were assessed across this range of network densities. More specifically, we calculated the following global network measures: global efficiency (the reciprocal of the node’s shortest path lengths to every other node), local efficiency (the reciprocal of a node’s shortest path length in the subgraph of the node’s neighbours), modularity (the extent to which a network can be divided into distinct modules), transitivity (the fraction of a node’s neighbours that are neighbours of each other), and betweenness centrality (the number of shortest paths in the network that traverse a given node; (Rubinov and Sporns 2010)). To pinpoint specific nodes whose status in the network differed between sexes, we additionally computed a range of nodal network measures: nodal global efficiency, nodal local efficiency, and betweenness centrality. While multiple network measures are available (Rubinov and Sporns 2010), we based our choice on the measures that have been reported to be more stable (Mårtensson et al. 2018).

While the modularity measure allows the quantification of the degree to which distinct nodes aggregate into more densely connected modules, it does not allow any insights on the topology of these modules. Therefore, to also describe modules in each of the subgroups qualitatively, we conducted modular analyses on the weighted correlation matrices using the Newman algorithm (Newman 2004).

All network analyses were conducted in Matlab R2019b using customized scripts based on the Brain Connectivity Toolbox version 2019-03-03 (Rubinov and Sporns 2010).

### Statistical Analysis

Group differences in demographic and clinical variables were checked with t-tests and Fisher’s exact tests for between-group comparisons of continuous and categorical variables, respectively. An α-level of *p* < 0.05 (two-tailed) denoted statistical significance. Between-group comparisons of network measures were conducted through 10000 nonparametric permutations at a range of network densities (20% – 50%, in steps of 1%). Again, the significance threshold was set to *p* < 0.05 for global network measures. Additionally, a false discovery rate (FDR) adjustment for multiple comparisons was applied at *p* < 0.05 (two-tailed) for nodal network measures at all network densities. Nodal measures surviving FDR correction for ≥ 5 network densities were considered significant.

## Results

### Cohort Characteristics

Overall, DLB patients and the age- and sex-matched healthy controls were comparable (Table 1). As expected, DLB patients and healthy controls differed in their MMSE scores, with DLB patients showing more cognitive impairment. DLB patients and healthy controls also differed in years of education, with male healthy controls having received a longer education. As expected, females had a lower intracranial volume than males. In the DLB patient group, male patients were more likely to exhibit parkinsonism with none of the other core features showing sex differences. There were no differences in AD co-pathology between female and male DLB patients.

### Sex differences in grey matter networks of healthy controls and DLB patients

**Figure 1:**
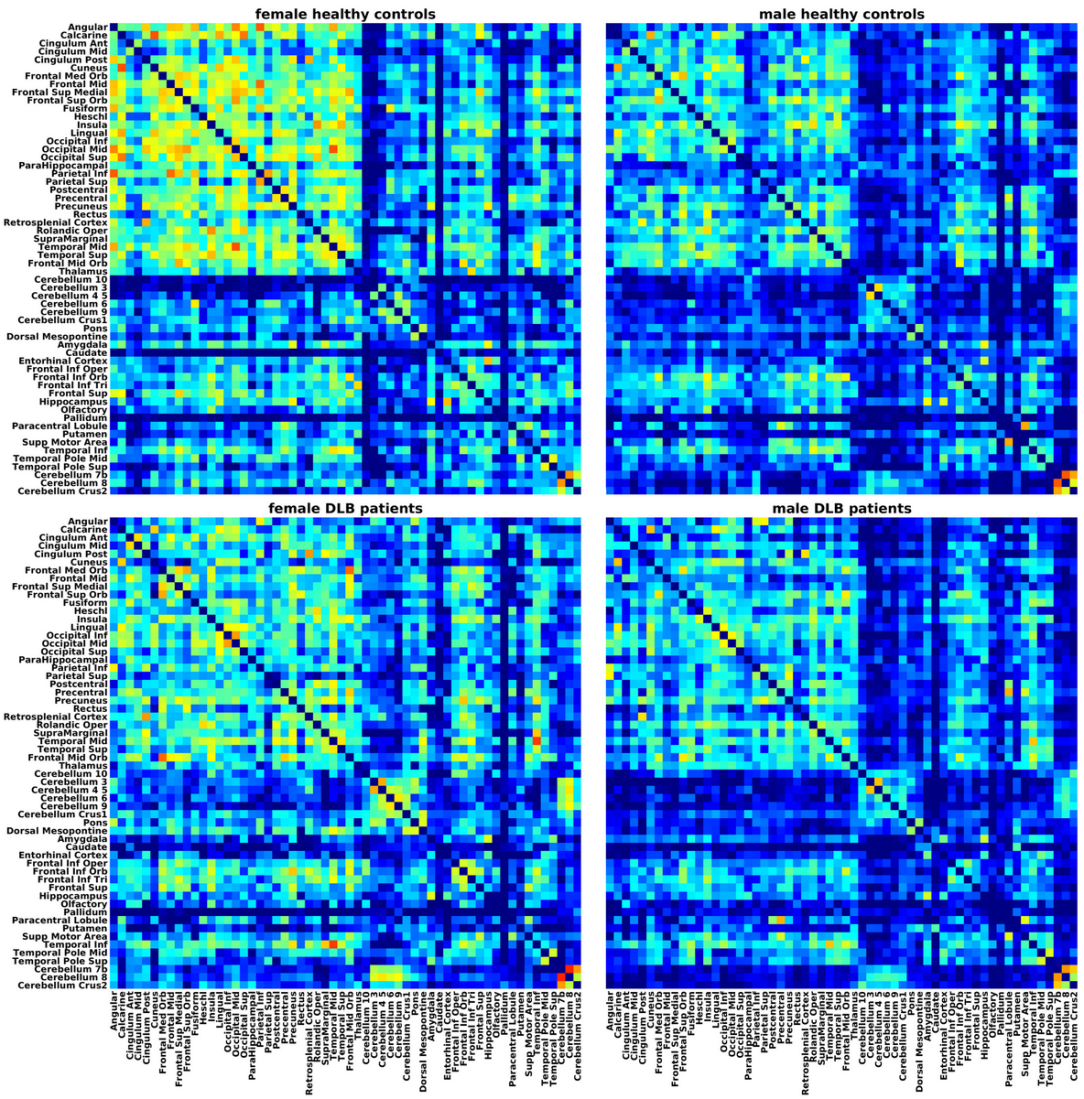
Grey matter networks. Weighted correlation matrices divided by diagnostic group and sex.

Visual inspection of the weighted correlation matrices (Fig. 1) showed that grey matter networks of DLB patients and healthy controls mainly differed between sexes and to a lesser degree across diagnostic groups. Specifically, male DLB patients and male healthy controls exhibited a more sparsely connected structural network compared to their respective female counterparts. This is reflected in the lower nodal strength in male DLB patients compared to female DLB patients (*t*_(114)_ = 4.28, *p* < 0.001) as well as in male healthy controls compared to female healthy controls (*t*_(114)_ = 4.52, *p* < 0.001).

**Figure 2:**
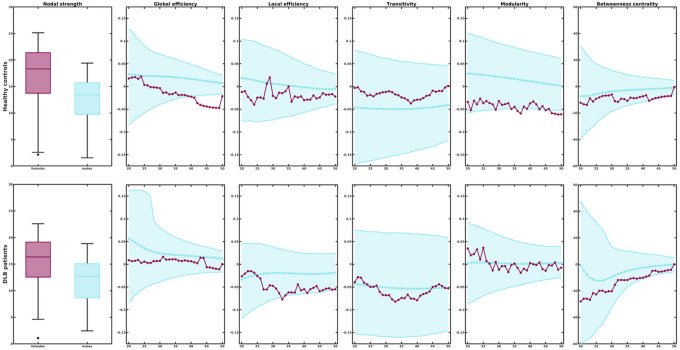
Comparison of global network measures between females and males in healthy controls and DLB patients. Network densities are displayed on the x-axis from min = 20% to max = 50%, in steps of 1%. Sex differences are displayed on the y-axis with 95% confidence intervals. Negative differences indicate lower value in females compared to males. Positive differences indicate higher values in females compared to males.

In healthy controls, sex differences emerged in global network measures. Especially, at higher network densities, males showed a higher global efficiency (Fig. 2). No sex differences emerged in local efficiency and transitivity in healthy controls. Moreover, compared to female healthy controls, males showed a higher modularity as well as a trend towards higher betweenness centrality.

In contrast, when comparing female and male DLB patients, none of the global measures differed significantly between sexes indicating that the expected sex differences found in the healthy population are lost in DLB patients.

None of the nodal network measures survived FDR-adjustment for any node in both healthy controls and DLB patients.

### Modules

**Figure 3:**
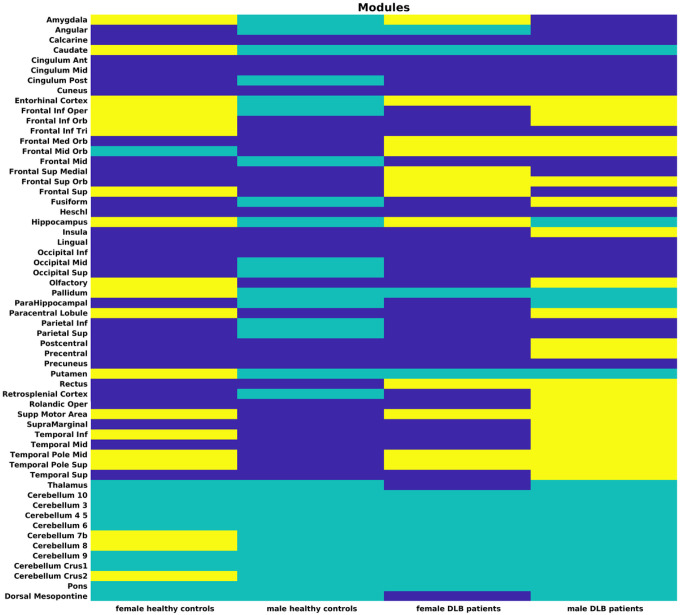
Modules for females and males in healthy controls and DLB patients. Module I in dark blue, module II in green, module III in yellow.

The sex-specific modules of healthy controls and DLB patients are illustrated in Fig. 3.

The grey matter network of female healthy controls split into 3 modules. Module I covered large parts of the cortex, including the superior frontal and temporal lobes, most occipital and parietal regions and the entire cingulum. In contrast, module II included only one cortical region, the middle orbital frontal gyrus, in combination with thalamus, the two pontine regions, and many cerebellar regions. Module III consisted of inferior frontal, olfactory cortex, two central, and inferior temporal regions, entorhinal cortex, almost all subcortical regions (except thalamus), as well as the remaining three cerebellar regions. Of note, female healthy controls were the only group in which cerebellar regions were assigned to more than one cluster.

Male healthy controls were the only group with only two modules, which consisted of 29 regions each. Module I included most frontal regions, including the olfactory cortex, the anterior and mid cingulum, regions surrounding the central sulcus, all temporal regions, inferior occipital regions, and insula. Module II combined most posterior brain regions of the parietal and occipital lobes with all subcortical, cerebellar and pontine regions.

In female DLB patients, module I encompassed inferior frontal, including olfactory cortex, most temporal, parietal, and occipital regions, while also including thalamus, and dorsal mesopontine. Female DLB patients were the only group in which a pontine region, specifically the dorsal mesopontine region, was associated with a different cluster than the majority of the cerebellar regions. Module II spanned subcortical regions (caudate, pallidum, putamen), posterior cingulum, all cerebellar regions and pons. The only cortical region included in module II was the angular gyrus. Module III combined olfactory cortex, orbitofrontal, superior frontal and superior temporal regions.

In male DLB patients, the grey matter network was also split into three modules of relatively similar size. Module I covered several frontal and parietal regions, the entire occipital lobe as well as amygdala and entorhinal cortex. Module II was an exclusively non-cortical module, consisting of caudate, hippocampus, parahippocampus, pallidum, putamen, thalamus, the posterior part of the cingulum, all cerebellar, and pontine regions. Lastly, module III included olfactory cortex, temporal and central regions, surrounding the central sulcus including rostral parietal regions, entorhinal cortex, and insula.

### DLB-specific sex differences in grey matter networks

**Figure 4:**
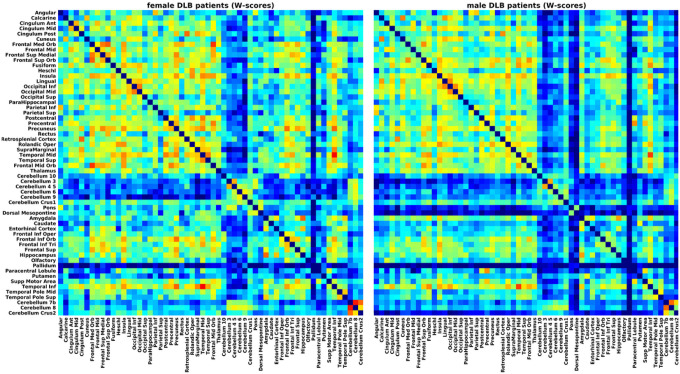
Grey matter networks. Weighted correlation of female and male DLB patients (W-scores).

**Figure 5:**
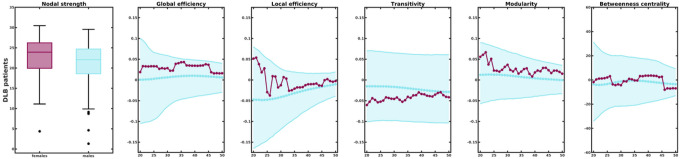
Comparison of global network measures between female and male DLB patients (W-scores). Network densities are displayed on the x-axis from min = 20% to max = 50%, in steps of 1%. Sex differences are displayed on the y-axis with 95% confidence intervals. Negative differences indicate lower value in females compared to males. Positive differences indicate higher values in females compared to males.

After W-scoring, the DLB-specific correlation matrices were more connected overall in both female and male DLB patients (Fig. 4). No significant sex differences in nodal strength arose specific to DLB (t_(114)_ = 1.95, p = 0.053, Fig. 5). DLB-specific grey matter networks did not significantly differ between sexes in any of the global or nodal network measures.

### Modules

**Figure 6:**
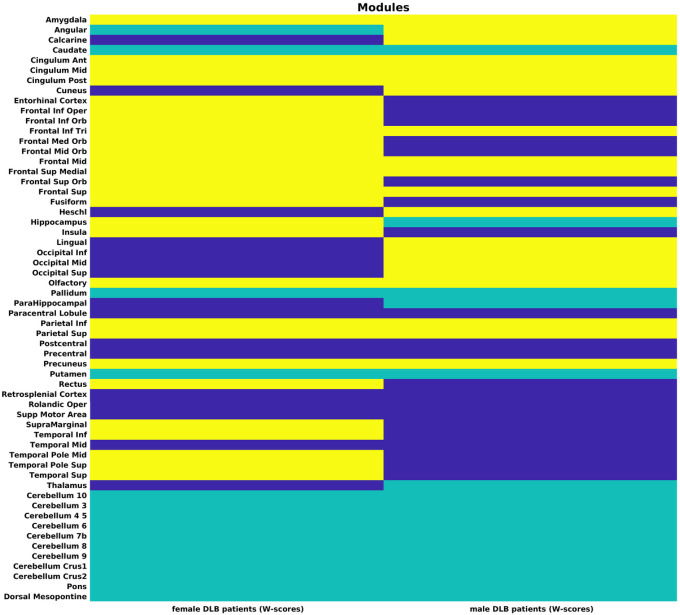
Modules for female and male in DLB patients (W-scores). Module I in dark blue, module II in green, module III in yellow.

Grey matter networks calculated on W-scores of both female and male DLB patients were divided into 3 modules (Fig. 6).

In female DLB patients, module I combined central and middle temporal regions, with all occipital regions, and thalamus. Module II included caudate, pallidum, putamen, all cerebellar and pontine regions as well as the angular gyrus. The largest module III spanned all frontal regions, including the olfactory cortex, cingulum, most temporal, and many parietal regions together with amygdala and hippocampus.

In W-scored data of male DLB patients, module I incorporated inferior and orbitofrontal regions, regions surrounding the central gyrus, most temporal regions, insula, and entorhinal cortex. Module II comprised most subcortical regions, including caudate, pallidum, putamen, thalamus, hippocampus, and parahippocampus in combination with all cerebellar, and pontine regions. In Module III, amygdala and cingulum clustered together with several frontal, including the olfactory cortex, parietal, and all occipital regions.

## Discussion

In this study we show that sex differences in grey matter networks in elderly depend on diagnostic group in so far as sex differences in global network measures materializing in healthy elderly individuals disappeared in DLB patients.

In a first step, we evaluated sex differences in grey matter networks in healthy elderly adults. Our analyses showed a lower network strength in male healthy controls, in combination with a higher global efficiency, higher modularity, and lower number of modules in male grey matter networks compared to female healthy controls. In other words, while grey matter network of male healthy controls were less connected, connectivity between brain regions was achieved more effectively according to small-world principles of neural networks (Strogatz 2001; Stam 2010). On the surface our findings are at odds with two cross-sectional studies, which considered healthy individuals from childhood/young adulthood to old age (Statsenko et al. 2022; Canales-Rodríguez et al. 2021). The first study found a higher vulnerability to agerelated effects on the microstructure level in men (Canales-Rodríguez et al. 2021) while the second study indicated that grey matter atrophy starts earlier and proceeds at a faster rate in males (Statsenko et al. 2022). However, our findings align with a study which considered exclusively older healthy participants similar to our cohort (Sang et al. 2021). This study revealed that women experience a steeper decrease in grey matter volume than males with more areas showing atrophy with increasing age in women compared to men. It has been suggested that the loss of the female advantage at older age is due to the decline of female sex hormone levels, especially estradiol, at menopause (Mosconi et al. 2021; Bustamante-Barrientos et al. 2021). In this process, the neuroprotective effect attributed to estradiol is decreasing, making post-menopausal women more vulnerable to neuronal stress, which has also been connected to a higher deposition of amyloid-β (Mosconi et al. 2021). Hence, given the advanced age of the participants in our study, it is plausible that the neuroprotective effect of estradiol was strongly attenuated if not completely expired, thus driving the degenerative process in female healthy controls to catch up and even overtake the neurodegeneration in male healthy controls, as reflected in the less efficient grey matter network topology in female healthy controls.

In contrast, almost none of the observed of sex differences in global network measures in healthy controls was significant in DLB patients. At a first glance, this might seem to be at odds with the previously reported sex differences in many aspects of DLB, ranging from epidemiology to pathogenesis to progression to symptom manifestation (Perez et al. 2010; Fereshtehnejad et al. 2013; Savica et al. 2013; Kane et al. 2018; Fujimi et al. 2008; van de Beek et al. 2020; Utsumi, Fukatsu, and Hara 2021; Tsunoda et al. 2018). However, even though the cross-sectional design of our study does not allow us to observe the effects directly, it marks a potentially disease-driven convergence of female and male grey matter networks in DLB. This may be driven by a more disruptive effect of DLB on the grey matter networks in male DLB patients. In fact, higher network strength observed in females compared to males in both healthy controls and DLB patients indicates a more connected grey matter network in females, aligning with a more widespread atrophy pattern. The accelerated deterioration of brain structure and function in women as compared to men was suggested to be even more pronounced in the advent of prodromal and clinical Alzheimer’s disease (Beheshti et al. 2021; Hua et al. 2010).

Since DLB, similar to other neurodegenerative diseases, can be considered to have an additive effect on normal ageing, we also investigated DLB-specific effects by calculating W-scores. In the W-scored data of DLB patients, females did not show a higher network strength than males, hence the sex difference in network strength seems to have been carried over from normal ageing and is not specific to DLB. Similarly, sex differences in dynamic functional connectivity measures derived from resting state fMRI from healthy controls were not evident in patients with Parkinson’s disease (Diez-Cirarda et al. 2021). Therein, the authors theorize that neurodegenerative processes of Parkinson’s override sex differences, which also seems a plausible explanation for the findings of our study. Indeed, disregarding sex differences, early changes in grey matter networks were shown in preclinical stages of Alzheimer’s dementia. More precisely, cognitively normal elderly with higher amyloid load showed more random grey matter network topology (ten Kate et al. 2018; Voevodskaya et al. 2018). More random grey matter network topologies were also associated with a steeper cognitive decline in individuals with subjective cognitive decline (Verfaillie et al. 2018) and prodromal dementia patients (Tijms et al. 2018; Dicks et al. 2018; Pelkmans et al. 2022). Apart from having a predictive value for disease progression, abnormal network organization has also been reported as a feature in Alzheimer’s disease and DLB patients with differences to healthy controls arising across a variety of global and nodal network measures (Pereira et al. 2016; Nicastro et al. 2021).

Different from a study by Nicastro and colleagues (Nicastro et al. 2021) in which grey matter networks strikingly mirrored large-scale resting-state networks, there no such clear delineation materialized in the modules of any subgroup. Other than the study by Nicastro and colleagues, in which grey matter networks were constructed exclusively based on correlations between cortical ROIs, in our study we made a point of additionally including subcortical, cerebellar, and pontine ROIs. According to the unified staging system in Lewy body diseases, the α-synuclein pathology originates in the olfactory bulb from where it first spreads to brain stem and limbic structures before reaching neocortical regions in stage IV (Beach et al. 2009). In turn, the accumulation of α-synuclein pathology in a specific brain region has been connected to neurodegeneration in this region (Simon et al. 2021), emphasizing the importance of including these regions with early accumulation of α-synuclein in our grey matter networks. This could be the reason why the modules in our study principally outlined the distinction between cortical and subcortical regions with the latter often clustering together with cerebellar and pontine regions. While this separation between cortical and non-cortical regions into different modules was also evident in healthy controls, specifically men, male DLB patients showed an even clearer separation by having one completely non-cortical volume. This points towards similar rates of atrophy in structures that are first affected by the Lewy body pathology (Beach et al. 2009). Another module in male DLB patients mainly combined frontal and posterior brain regions. The concerted decline of these brain regions is in line with functional disruptions in frontoparietal connections which were repeatedly reported in DLB patients (Peraza, Taylor, and Kaiser 2015; Peraza et al. 2014; Dauwan et al. 2016). The more disjointed combination of brain regions within and across modules, particularly the uncoupling of cerebellar regions in female healthy controls and the separation of pontine regions in female DLB patients aligns with the observed higher network strength in females of both diagnostic groups, both indicating a less distinct atrophy pattern in females. Unlike in male DLB patients, the topology of network disruptions we observed in female DLB patients does not overlap as consistently with brain regions that have been shown to accumulate α-synuclein early in the disease process. Potentially, this less DLB-specific network disruption in female patients may be attributable to co-pathologies. Specifically, Alzheimer’s disease co-pathology was previously more often found in female than male DLB patients (Barnes, Lamar, and Schneider 2019; Ferreira et al. 2020). However, comparing the positivity in both β-amyloid and tau biomarkers indicated no significant sex differences in Alzheimer’s disease co-pathology in our DLB cohort, making a sex-specific impact on grey matter networks improbable.

Slight discrepancies between our findings and previous results, that showed more randomized and/or disrupted grey matter networks in individuals undergoing cognitive decline (Nicastro et al. 2021; He, Chen, and Evans 2008; Tijms et al. 2018; Dicks et al. 2018; Pelkmans et al. 2022), may be related to differences in the construction of the respective grey matter networks. Among many methodological choices the parcellation scale demonstrably influences network measures (Qi et al. 2015; Zalesky et al. 2010). Even though the number of nodes in our grey matter networks was very similar to previous studies conducted in DLB and Alzheimer’s disease (Nicastro et al. 2021; Pereira et al. 2016; He, Chen, and Evans 2008) differences may have arisen due to our more comprehensive inclusion of non-cortical brain regions. More precisely, other studies focussed on cortical regions with the occasional inclusion of subcortical regions, while we additionally incorporated cerebellar and pontine regions. Moreover, most previous studies based their grey matter networks on covariations of regional cortical thickness and occasionally volumes of subcortical structures (Nicastro et al. 2021; Pereira et al. 2016; He, Chen, and Evans 2008). In contrast, we opted for a more consistent approach by exclusively considering the volumes of all included brain regions. While different morphometric parameters are associated with each other during the ageing process, their specific interactions may vary across different brain regions thus resulting in distinctive network topologies depending on which measure was employed (Lemaitre et al. 2012; Sanabria-Diaz et al. 2010). Lastly, it needs to be considered that grey matter networks are based on Pearson correlation coefficients which are stabilizing with increasing sample size (Schönbrodt and Perugini 2013; Bates et al. 1996). Our sample included double to ten times the number of DLB patients included in previous studies that investigated the sex differences on structural MRI measures in DLB (Abdelnour et al. 2020; Ballmaier et al. 2004). Nonetheless, women were still significantly underrepresented in line with the male predominance in most European and North American DLB cohorts, from which we drew our sample (Perez et al. 2010; Fereshtehnejad et al. 2013; Savica et al. 2013; Kane et al. 2018). Consequently, the smaller size of the female subsamples may be partially responsible for the more random network structure of their grey matter networks.

In this study, we compared, for the first time, sex differences in grey matter networks of DLB patients and elderly healthy controls from large international cohorts. We demonstrated a discrepancy in the presence of a global impact of sex on the grey matter networks in healthy individuals and the absence of most of these sex differences in grey matter networks in DLB patients. The main limitation of our study is its cross-sectional design and the associated lack of longitudinal assessments. Hence, we cannot determine when these differential sex effects first alter the grey matter networks and how changes develop over time. Longitudinal assessments in future studies should preferably cover the entire interval from preclinical stages to disease manifestation, including progression into more severe disease stages, in DLB.

Simultaneously following the sex-dependent alterations in grey matter networks of elderly healthy controls during normal ageing would allow us to directly observe at which point trajectories in female and male DLB patients diverge from the trajectories of age-matched controls of the same sex. Apart from identifying the starting point of the disease-mediated bifurcation within one sex, longitudinal assessments of grey matter networks would allow us to monitor the steepness of trajectories depending on age and diagnostic group. In turn, this would give us insights into at which stage grey matter networks of male and female DLB patients converge again, a process that might be driven by an asymmetric impact of the disease on patients of one sex in particular.

To sum up, global network measures and network strength indicated subtle sex effects in the topology of grey matter networks in elderly healthy controls. These sex effects were mitigated in DLB patients, indicating a sex-specific vulnerability of the brain network to the neurodegenerative processes in DLB. This disappearance of sex effects in DLB patients aligns with results from a study functional connectivity in Parkinson’s disease (Diez-Cirarda et al. 2021) and future studies should investigate whether this feature is specific to α-synucleinopathies. Our results also underline the importance of considering the patient’s sex in future precision medicine approaches both in delaying normal and pathological ageing processes. While integrating sex as a factor in all clinical processes is important, it is especially crucial in diseases like DLB, in which sex differences permeate many aspects of the disease and may thus play a central role in the development of treatment strategies.

## Figures and Tables

**Table 1:** Demographic and clinical characteristics of DLB patients and healthy controls. For continuous variables data is provided as mean ± standard deviation.

	DLB patients (*n* = 165)		Healthy controls (*n* = 165)		
Variables	Female (n = 45)	Male (n = 119)	Female (n = 45)	Male (n = 119)	Statistics
Age- (years)	69.9 ± 9.1	68.7 ± 8.4	69.9 ± 9.1	68.7 ± 8.4	n.s. (ANOVA)
Education (years)	13.4 ± 3.5	13.7 ± 4.0	13.9 ± 3.0	14.5 ± 3.5	p **< 0.001** (ANOVA)
Disease duration (years)	4.8 ± 3.1	5.8 ± 4.6	n.a.	n.a.	n.s. (t-test)
MMSE	24.4 ± 4.2	22.3 ± 5.5	26.5 ± 3.68	25.4 ± 5.0	p **< 0.001** (ANOVA)
TIV	1439.8 ± 117.7	1632.0 ± 135.0	1415.4 ± 114.6	1622.0 ± 129.9	p **< 0.001** (ANOVA)
Parkinsonism	35 (77.8%)	106 (90.6%)^a^	n.a.	n.a.	p **= 0.035** (t-test)
Visual hallucinations	27 (60.0%)	62 (53.4%)^b^	n.a.	n.a.	n.s. (t-test)
Cognitive fluctuations	36 (81.8%)^c^	94 (83.9%)^d^	n.a.	n.a.	n.s. (t-test)
RBD	27 (71.8%)^d^	89 (80.2%)^e^	n.a.	n.a.	n.s. (t-test)
AD co-pathology	10 (11.8%)^e^	3 (8.1 %)^f^	n.a.	n.a.	n.s. (t-test)

Missing data for ^a^*n* = 2, ^b^*n*=3, ^c^*n*=1, ^d^*n*=7, ^e^*n* = 8, ^f^*n* = 34 DLB patients.
